# Real-World Effectiveness of DIMS Spectacle Lenses for Myopia Control in a Turkish Pediatric Population: A Retrospective Study Using Age-Specific Physiological Growth Curves

**DOI:** 10.3390/children12111435

**Published:** 2025-10-23

**Authors:** Nilay Akagun, Ugur Emrah Altiparmak

**Affiliations:** Department of Ophthalmology, Acıbadem Hospital Ankara, Yukarı Dikmen Tevfik Kıs St. No: 6, Çankaya/Ankara 06450, Turkey; emrah.altiparmak@gmail.com

**Keywords:** myopia, myopia control, DIMS spectacle lenses, physiological growth curves, children, Turkey

## Abstract

Background/Objectives: This study aimed to evaluate the one-year real-world effectiveness of Defocus Incorporated Multiple Segments (DIMS) spectacle lenses in controlling myopia progression in a Turkish pediatric cohort and to identify predictors of treatment response using age-specific physiological growth curves. Methods: This retrospective single-center study included 54 patients (108 eyes) aged 6–16 years with myopia who wore DIMS spectacle lenses full time for 12 months. The primary outcomes were spherical equivalent refraction (SER) success and axial length (AL)-based treatment response. Treatment success was defined as an SER progression of ≤0.50 dioptres per year and AL elongation within age-specific physiological limits. Subgroup analyses were performed according to age, gender, and the baseline AL group. Results: After 12 months, the mean AL elongation was 0.14 ± 0.31 mm, and the mean SER change was −0.28 ± 0.42 D. SER-based success was achieved in 85.2% of eyes. For AL-based response, 61.1% of eyes showed a good response, 16.7% showed a low–moderate response, and 22.2% had no response. Eyes with moderate baseline AL exhibited significantly less axial elongation than those with high baseline AL (*p* = 0.001). Children aged ≥ 10 years demonstrated better AL-based responses (*p* = 0.016). The baseline AL group significantly predicted the AL treatment response, while both the baseline AL group and gender predicted SER success. Gender was associated with SER outcomes but not with AL-based response. Conclusions: DIMS spectacle lenses effectively reduced myopia progression and axial elongation in this real-world Turkish pediatric cohort. Baseline AL and gender were significant predictors of treatment outcomes. Incorporating age-specific physiological growth curves provided an individualized framework for interpreting treatment success. Further prospective studies with larger samples and longer follow-up are warranted to confirm these findings.

## 1. Introduction

Myopia has emerged as a leading cause of visual impairment in children and adolescents worldwide, with its prevalence projected to reach epidemic proportions by 2050, affecting nearly half of the global population [1]. This progressive refractive condition, which typically begins in early school years, is primarily driven by abnormal axial elongation and is associated with an increased lifetime risk of severe ocular complications, including retinal detachment, myopic maculopathy, glaucoma, and choroidal neovascularization [2,3]. Even low levels of myopia have been shown to increase these risks, which rise exponentially with greater axial elongation [4,5].

In recent decades, a sharp rise in myopia prevalence has been documented not only in East Asia but also in Western countries, including Turkey and other European nations, largely attributable to environmental and behavioral changes such as increased near work and reduced outdoor time [6,7]. Given the growing public health burden of myopia-related morbidity, early and effective control of axial elongation has become a central goal in pediatric ophthalmology [8,9].

Current strategies to slow myopia progression include pharmacological interventions, such as low-concentration atropine eye drops, as well as optical approaches, including multifocal contact lenses and spectacle lenses designed to induce myopic defocus [10,11,12].

Among these, Defocus Incorporated Multiple Segments (DIMS) spectacle lenses represent a promising innovation, combining a central single-vision correction zone with concentric rings of plus-powered lenslets to create simultaneous myopic defocus across the retina [13]. This design has demonstrated significant efficacy in reducing both spherical equivalent progression and axial elongation in multi-year randomized trials [14] and has been further validated through subsequent analyses [15]. DIMS lenses have been available in Turkey since June 2023 under the brand name Miyosmart^®^ (Hoya^®^ Lens Thailand Ltd., Bangkok, Thailand).

Although the efficacy of DIMS lenses has been well established in Asian populations, limited evidence exists regarding their real-world effectiveness in European cohorts, where physiological ocular growth trajectories and baseline refractive characteristics may differ. Furthermore, the influence of baseline factors such as axial length, refractive error, age, and gender on treatment response remains incompletely understood, despite their potential to guide personalized treatment strategies [16,17].

In this context, the present study aimed to evaluate the one-year real-world effectiveness of Defocus Incorporated Multiple Segments (DIMS) spectacle lenses in slowing myopia progression in a Turkish single-center pediatric cohort aged 6 to 16 years. Treatment outcomes were evaluated based on spherical equivalent refraction (SER)-based treatment success and axial length (AL)-based treatment response, with subgroup analyses conducted according to age group, gender, baseline SER, and baseline AL. Axial elongation was additionally interpreted relative to age-specific physiological growth curves to provide a more precise and individualized assessment of treatment response in clinical practice.

## 2. Methods

### 2.1. Study Design

This retrospective observational study was conducted from 1 May 2023 to 31 May 2024 at the Department of Ophthalmology, Acibadem Hospital, Ankara, Turkey. The study was performed in accordance with the Declaration of Helsinki and was approved by the Acibadem University Medical Research Ethics Committee (ATADEK) on 8 May 2025 (approval number 2025-07/294). Written informed consent was obtained from all participants and their legal guardians prior to inclusion in the study.

### 2.2. Inclusion and Exclusion Criteria

Inclusion criteria included

Age between 6 and 16 years;
Diagnosis of myopia with cycloplegic spherical equivalent refraction (SER) ≤ −0.50 D;
Anisometropia < 1.50 D;
Astigmatism < 2.00 D;
No history of prior myopia-control treatment.

Exclusion criteria were

Presence of ocular pathology (e.g., glaucoma, cataract, keratopathy, amblyopia, or strabismus);
Systemic diseases or conditions;
Any ocular findings that could influence optical measurements or treatment adherence.

### 2.3. Intervention and Data Collection

All participants had been prescribed Defocus Incorporated Multiple Segments (DIMS) spectacle lenses (Miyosmart^®^, Hoya Lens Thailand Ltd., Bangkok, Thailand) for full-time wear, except during sleep or bathing. Parental supervision of treatment adherence was routinely recorded in clinical files based on questioning during follow-up visits, where parents reported average daily wear time and any significant deviations from full-time use. In total, 54 patients (108 eyes) who met the eligibility criteria were included. Patient records contained age, gender, visit dates, cycloplegic spherical equivalent refraction (SER), and axial length (AL) measurements.

Cycloplegic autorefraction and axial length measurements were performed as part of routine myopia follow-up examinations and were available in the medical records. Cycloplegic refraction was assessed 30 min after administering two drops of 1% tropicamide (Tropamid^®^, Bilim İlaç, İstanbul, Turkey) at 5 min intervals, following standard clinical protocol in non-strabismic pediatric patients [18]. Measurements were obtained using a Topcon CKR-8900 autorefractometer (Topcon Corp., Tokyo, Japan) and repeated until three consecutive readings demonstrated a standard deviation of less than 0.05 D.

Axial length had been measured using the Zeiss^®^ IOL Master 700 (Carl Zeiss Meditec AG, Jena, Germany), with values recorded once the standard deviation was less than 0.05 mm. These procedures reflect routine clinical practice and follow published recommendations for measurement repeatability and agreement in ophthalmology [19].

### 2.4. Outcomes

Treatment outcomes were evaluated using two distinct criteria: spherical equivalent refraction (SER)-based success and axial length (AL)-based treatment response. The primary outcomes were SER-based treatment success and AL-based treatment response over the 12-month follow-up period. The AL-based treatment response was determined based on the Axial Myopia Management Classification (AMMC) system developed by Kaymak et al. [20].

SER-based success: Defined as an annual myopia progression of no more than −0.50 diopters (D). A cut-off value of −0.50 D per year was chosen to define treatment success, as this threshold is widely regarded as clinically meaningful for identifying significant myopia progression in children and has been adopted in previous myopia control studies [13,21].

AL-based treatment response: Evaluated relative to age-specific physiological axial growth rates, based on normative growth curves from Truckenbrod et al. [21] and Graff et al. [15]. Each eye was categorized as follows:

Good response: Axial elongation within the physiological range expected for the child’s age.

Low response: Axial elongation slightly above the age-specific physiological range. “Slightly above the physiological range” was defined as within 0.05–0.10 mm/year of the upper limit of age-specific physiological elongation, whereas “markedly above” was defined as greater than 0.10 mm/year above this range [15,21].

No response: Axial elongation markedly above the physiological range.

The physiological axial-length growth rates were derived from the normative curves reported by [15,22]. In these models, expected elongation decreases from approximately 0.25–0.30 mm per year at age 6 to 0.10–0.15 mm per year at age 15, reflecting the natural slowing of ocular growth with age. These outcomes were analyzed using generalized linear mixed models (GLMMs) with a random intercept for child to account for the correlation between both eyes of the same participant. SER-based treatment success was modeled as a binary outcome, while AL-based response was treated as an ordinal outcome across the three categories. Fixed effects included gender, age group, and baseline AL group. Statistical significance was defined as *p* < 0.05, with exact *p*-values reported unless <0.001.

### 2.5. Subgroup Analyses

Subgroup analyses were conducted to examine the influence of baseline characteristics on treatment outcomes:

Age group: 6–10 years vs. 11–16 years [23].

Baseline AL group: Moderate (<98th percentile) vs. high (>98th percentile) based on Truckenbrod et al. [22].

Gender: Male vs. female.

### 2.6. Statistical Analysis

All statistical analyses were performed using IBM SPSS Statistics version 29.0 (IBM Corp., Armonk, NY, USA). Continuous variables were summarized as mean ± standard deviation (SD) or median (range), and categorical variables as frequencies and percentages. The Shapiro–Wilk test was used to assess normality.

Because both eyes of each participant were included, Generalized Estimating Equations (GEE) were applied to account for intra-subject correlation. A binary GEE model with robust standard errors was used to analyze SER-based treatment success, and an ordinal GEE model was applied to evaluate the three-level AL-based treatment response (good, low, no). This population-averaged modeling approach allowed valid inference without assuming independence between eyes, providing more reliable estimates of predictive factors.

### 2.7. Power Analysis

A post hoc power analysis was conducted using G*Power 3.1 software (Faul et al., 2009) [24] to confirm the adequacy of the sample size for binary comparisons. For SER-based treatment success (85.2% vs. 14.8%), the calculated statistical power exceeded 0.99, while for AL-based treatment response (64.8% vs. 35.2%), the power was 0.9999 (α = 0.05). These results indicate that the study was sufficiently powered to detect statistically significant effects for the outcomes analyzed.

## 3. Results

Among all eyes, 56 (51.9%) were from female and 52 (48.1%) from male participants. Based on age distribution, 68 eyes (63.0%) were from children aged < 10 years and 40 eyes (37.0%) from those aged ≥ 10 years. According to age-specific reference values, 60 eyes (55.6%) were classified as having moderate baseline AL and 48 eyes (44.4%) as high baseline AL. The baseline demographic and ocular characteristics of the study population are summarized in Table 1a,b.

After 12 months of DIMS lens wear, SER treatment success—defined as an annual SER change ≤0.50 D—was achieved in 81.5% (88 eyes), while 18.5% (20 eyes) were classified as unsuccessful.

**Figure 1:**
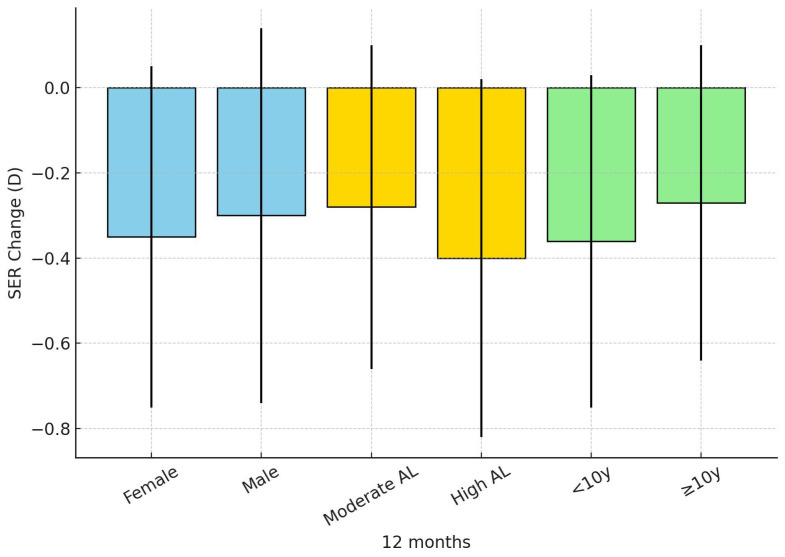
SER change over 12 months by subgroup. Legend: Subgroups are categorized by gender (female, male), baseline axial length (moderate AL, high AL), and age group (<10 years, ≥10 years). Bars represent mean values, and error bars indicate standard deviations.

Figure 1 illustrates the changes in spherical equivalent refraction (SER) over 12 months according to gender, baseline axial length, and age subgroups.

AL treatment response, categorized as good, low–moderate, or no response according to axial elongation, was observed as follows: 66 eyes (61.1%) showed a good response, 18 eyes (16.7%) a low–moderate response, and 24 eyes (22.2%) no response.

**Figure 2:**
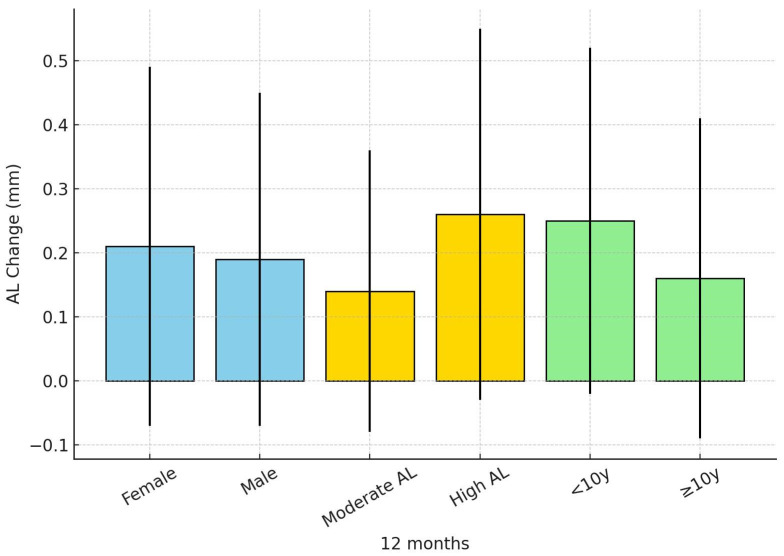
Axial length (AL) change over 12 months by subgroup. Legend: Subgroups are divided by gender (female, male), baseline axial length (moderate AL, high AL), and age group (<10 years, ≥10 years). Bars represent mean values, and error bars indicate standard deviations.

Figure 2 illustrates the axial length (AL) changes over 12 months according to gender, baseline AL, and age subgroups.

The distributions of SER success and AL response across gender, age, and baseline AL subgroups are shown in Table 2.

**Table 2:** a. SER Treatment Success According to Subgroups. b. AL Response According to Subgroups.

**a**					
**Variable**	**Category**	**Successful,** ***n*** **(%)**		**Unsuccessful,** ***n*** **(%)**	**Total (** ***n*** **)**
Gender	Female	41 (73.2%)		15 (26.8%)	56
	Male	47 (90.4%)		5 (9.6%)	52
Age Group	Young children (<10 years)	56 (82.4%)		12 (17.6%)	68
	Older children (≥10 years)	32 (80.0%)		8 (20.0%)	40
Baseline AL Group	Moderate baseline AL	53 (88.3%)		7 (11.7%)	60
	High baseline AL	35 (72.9%)		13 (27.1%)	48
Total		88 (81.5%)		20 (18.5%)	108
**b**					
**Variable**	**Category**	**Good Response,** ***n*** **(%)**	**Low–Moderate Response,** ***n*** **(%)**	**No Response,** ***n*** **(%)**	**Total (** ***n*** **)**
Gender	Female	33 (58.9%)	8 (14.3%)	15 (26.8%)	56
	Male	33 (63.5%)	10 (19.2%)	9 (17.3%)	52
Age Group	Young children (<10 years)	45 (66.2%)	12 (17.6%)	11 (16.2%)	68
	Older children (≥10 years)	21 (52.5%)	6 (15.0%)	13 (32.5%)	40
Baseline AL Group	Moderate baseline AL	43 (71.7%)	11 (18.3%)	6 (10.0%)	60
	High baseline AL	23 (47.9%)	7 (14.6%)	18 (37.5%)	48
Total		66 (61.1%)	18 (16.7%)	24 (22.2%)	108

Note: Data are expressed as the number and percentage of eyes within each subgroup. SER = spherical equivalent refraction; AL = axial length. All values represent descriptive distributions, as only one treatment group was included in the study.

**Figure 3:**
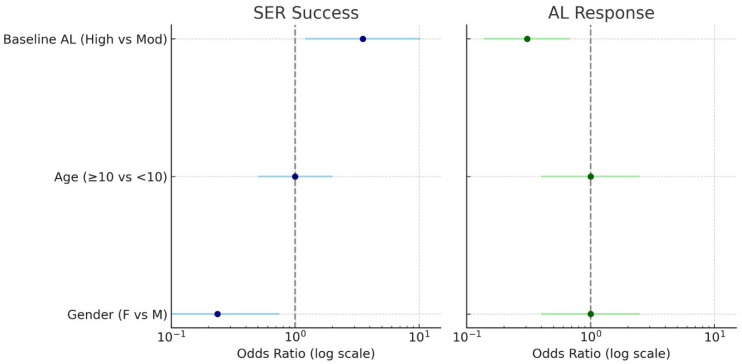
Predictors of SER success and AL-based treatment response. Legend: Forest plot illustrating the predictors of treatment outcomes. Forest plot illustrating the predictors of treatment response. Odds ratios (ORs) with 95% confidence intervals are shown on a logarithmic scale. SER-based treatment success (**left panel**) includes gender, baseline AL group, and age group as predictors, showing significant associations for gender and baseline AL group. AL-based treatment response (**right panel**) includes the same predictors, with only baseline AL group showing a significant association. The vertical dashed line at OR = 1 indicates no effect.

(AL) group as significant predictors of SER-based treatment success, whereas age group showed no independent association. Female participants had a lower likelihood of achieving SER success, while eyes with moderate baseline AL had a higher probability of success compared with those with high baseline AL (Table 3). For the AL-based treatment response, only baseline AL group was significantly associated with the outcome: eyes with high baseline AL showed a reduced likelihood of favorable axial elongation compared with those with moderate baseline AL, whereas gender and age group were not significant predictors (Table 3). These associations are summarized visually in the forest plot (Figure 3), which illustrates the odds ratios and 95% confidence intervals for all predictors included in the GEE models.

## 4. Discussion

After 12 months of DIMS lens wear, the mean axial elongation in our cohort was 0.20 ± 0.27 mm, and the mean change in SER was −0.32 ± 0.42 D. Both values were lower than those typically reported in East Asian cohorts, where annual axial elongation of 0.3–0.4 mm and SER progression of approximately −0.60 D have been observed [25]. These findings indicate that DIMS lenses achieve a comparable level of effectiveness in non-East Asian populations, including Turkish children, despite similar exposure to environmental risk factors such as intensive education and prolonged near work.

In our cohort, 85.2% of eyes achieved SER-based treatment success, defined as an annual progression of ≤0.50 D. Regarding the AL-based outcome, 61.1% of eyes showed a good response, 16.7% a low response, and 22.2% no response after 12 months of DIMS lens wear. Although younger children tended to exhibit greater axial elongation in subgroup analyses, the age group was not a significant independent predictor of treatment response in the multivariable model. Male participants demonstrated slightly longer baseline AL than females, consistent with previously reported gender-related biometric differences in ocular dimensions [4].

Graff et al. emphasized that assessing axial elongation relative to age-specific physiological growth curves provides a more accurate reflection of treatment efficacy than relying solely on absolute elongation values [15]. In their cohort, DIMS lens wearers exhibited an annual AL increase of approximately 0.10 mm, which closely matched the physiological growth rate observed in emmetropic children. In contrast, the mean elongation observed in our study (0.20 ± 0.27 mm, 95% CI: 0.15–0.25 mm) remained within the lower range of expected values for myopic progression and was substantially smaller than the elongation typically reported in untreated myopic eyes (0.30–0.40 mm per year or 0.50–0.75 D per year) [13,25]. These results suggest that DIMS lenses effectively reduce axial growth toward age-appropriate physiological levels, even in populations outside East Asia.

Physiological eye growth naturally declines with age, and younger children exhibit greater axial elongation even in the absence of progressive myopia. Therefore, interpreting AL changes without accounting for age-related variation may lead to misjudgment of treatment outcomes. In the present study, age-specific normative growth curves from Truckenbrod et al. [22] were applied, following the approach of Graff et al. [15], to evaluate treatment effects relative to physiological expectations. According to Graff et al., axial elongations that remain within or only slightly exceed age-specific physiological ranges (≤+0.1 mm above the upper limit) can still be interpreted as physiologically acceptable growth, whereas markedly exceeding values (>+0.1 mm) indicate pathologic progression beyond normal developmental variation. This method enabled an individualized assessment of AL growth in our cohort, where the mean elongation was 0.20 ± 0.27 mm, and 73.1% of eyes demonstrated responses (good or low) consistent with or only slightly exceeding age-appropriate physiological growth, providing an ethically acceptable alternative to untreated controls in a real-world clinical setting.

Brennan et al. [26] emphasized the importance of age-adjusted evaluations when interpreting treatment outcomes, as physiological eye growth varies with age. In our cohort, younger children exhibited greater axial elongation, consistent with normal physiological patterns; however, age group was not an independent predictor of treatment response in the multivariable GEE model applied in this study.

Our findings are consistent with those of Lam et al. [13,14,27], who demonstrated the sustained effectiveness of DIMS lenses in East Asian children over follow-up periods of up to six years. In their two-year randomized controlled trial, Lam et al. reported a mean annual axial elongation of 0.21 mm in the DIMS group, significantly lower than the 0.55 mm observed in the single-vision lens (SVL) group [13]. The three-year extension confirmed this suppression effect, with elongation rates decreasing to approximately 0.10 mm per year [14]. Furthermore, six-year longitudinal data demonstrated maintained efficacy without evidence of rebound following treatment discontinuation [27]. In comparison, the mean axial elongation observed in our cohort (0.20 ± 0.27 mm after one year) closely aligns with the results from Lam et al.’s two-year trial, supporting the effectiveness of DIMS lenses in slowing axial eye growth in our Turkish children cohort under real-world clinical conditions.

Similarly, Chamberlain et al. [21] reported that MiSight^®^ 1-day lenses reduced annual axial elongation to approximately 0.30 mm, a value close to the physiological growth observed in emmetropic children (~0.24 mm/year). In comparison, the mean axial elongation in our cohort (0.20 ± 0.27 mm) was lower than that reported with MiSight^®^, further supporting the efficacy of DIMS lenses in slowing axial eye growth.

Graff et al. [15] reanalyzed Lam et al.’s dataset and found that 65% of eyes in the DIMS group, compared with only 16% in the SVL group, exhibited axial elongation within age-matched physiological limits. In our cohort, 64.8% of eyes showed a good response (i.e., within or just above physiological limits), 8.3% showed a low response, and 26.9% had no response. These results align with Graff et al.’s findings. Moreover, similar to their subgroup analysis, our data indicated more favorable responses among eyes with moderate baseline AL and in older age groups, supporting the broader efficacy of DIMS lenses across baseline characteristics.

In a real-world German study, Neller et al. [16] evaluated DIMS lens effectiveness using age-specific physiological growth curves and reported a 46% success rate for axial length (AL) control and a 65% success rate for myopia progression when only within-physiological (good) responses were considered. In comparison, our cohort demonstrated higher overall effectiveness, with 64.8% of eyes achieving a good AL-based response, 8.3% a low response, and 26.9% showing no response. Additionally, SER-based treatment success was achieved in 85.2% of eyes, further supporting the strong refractive control effect of DIMS lenses in real-world conditions. Consistent with Neller et al., we observed more favorable outcomes in eyes with moderate baseline AL, in older age groups, and across gender subgroups. However, whereas age was a significant predictor in our analysis, Neller et al. reported generally lower success rates among younger children of both genders. Differences in success criteria, sample size, ethnicity, and baseline myopia severity may explain variations in response rates, yet collectively these findings reinforce the robust effectiveness of DIMS lenses across baseline AL, age, and gender subgroups.

In our subgroup analysis, differences in axial elongation and SER progression were examined using Generalized Estimating Equations (GEE), which appropriately account for the inclusion of both eyes per participant and the resulting intra-subject correlation. A binary GEE model was applied for SER-based treatment success, and an ordinal GEE model for the three-level AL-based response (good, low, or no response). This population-averaged modeling approach enabled robust inference without assuming independence between eyes, thereby providing more reliable estimates of predictive factors.

The results showed that baseline AL was the only significant predictor for both SER success (*p* = 0.021) and AL-based response (*p* = 0.004). Gender was significantly associated with SER success (*p* = 0.013), while age group showed no independent association in the multivariable models. Because both eyes were included, direct pairwise subgroup comparisons were not performed, as GEE inherently adjusts for within-subject correlations rather than testing independent group differences. The weak correlation between changes in spherical equivalent refraction (SER) and axial length (AL) likely reflects compensatory lens and corneal adjustments, as well as optical adaptation induced by DIMS lenses. While AL elongation indicates structural growth, refractive changes may be minimized by these mechanisms and by measurement differences between refraction and biometry [14,21,23].

Overall, these findings indicate that baseline ocular characteristics—particularly baseline AL—are the most decisive determinants of treatment outcomes. Once baseline differences are accounted for, the effects of age and gender become less consistent, suggesting that myopia progression is primarily driven by intrinsic ocular anatomy rather than demographic factors. This interpretation is consistent with the findings of Nucci et al. [17], who identified baseline AL as a major determinant of treatment outcomes. However, while Guimarães et al. [28] reported age as an independent predictor of treatment response, our analysis showed that age significantly influenced SER-based success but not AL-based outcomes, suggesting a differential role of age across refractive and biometric parameters.

Similar conclusions have been drawn in comparative studies of different myopia control interventions. Chia-Yi Lee et al. [29] and Lu et al. [30] compared orthokeratology and DIMS lenses, showing that higher baseline SER was consistently associated with greater axial elongation. However, their conclusions regarding baseline AL differed: Lu et al. [30] reported that higher baseline AL predicted slower elongation, whereas Lee et al. [29] found no significant association. In our study, baseline AL emerged as the most significant determinant of both SER success and AL-based treatment response, with moderate baseline AL associated with more favorable outcomes than high AL. These findings reinforce that baseline ocular anatomy—particularly axial length—plays a key role in modulating treatment efficacy across different interventions and populations.

Recent studies have also examined predictive factors for treatment efficacy in DIMS and highly aspherical lenslet (HAL) lenses. Guo et al. [31] identified younger age as the only significant predictor of axial elongation, while Lembo et al. [32] found no significant associations for age or baseline ocular parameters in a European pediatric cohort. In contrast, our findings highlight baseline AL as the key determinant of treatment outcomes, with eyes having moderate baseline AL showing less axial elongation than those with high AL. Although age was not an independent predictor in our GEE models, gender was associated with SER outcomes, with males achieving slightly higher treatment success. These results emphasize the variability of predictive factors across populations and optical designs, supporting the need for individualized myopia control approaches.

Across published studies, baseline predictors of myopia progression have shown inconsistent trends. Nucci et al. [17] linked higher AL to faster progression, Lu et al. [30] reported the opposite pattern, Guo et al. [31] emphasized younger age, and Lembo et al. [32] observed no significant predictors. Taken together, these findings suggest that while the influence of individual predictors varies across cohorts and study designs, baseline AL consistently remains the most reliable indicator of treatment response.

## 5. Strengths and Limitations

This study has several strengths. To our knowledge, it is the first to evaluate the real-world effectiveness of DIMS spectacle lenses in a Turkish pediatric cohort using age-specific physiological growth curves to enable individualized assessment. The inclusion of both eyes, with appropriate adjustment for intra-subject correlation through GEE modeling, strengthened the robustness of the analyses. Furthermore, subgroup evaluations by baseline AL and age provided valuable insights into predictors of treatment response, reinforcing the importance of a personalized approach to myopia management.

However, several limitations should be acknowledged. The retrospective, single-center design may limit generalizability. The one-year follow-up period precludes conclusions about long-term efficacy or potential rebound effects after discontinuation. Treatment compliance was based on parental reports rather than objective monitoring (e.g., wear-time sensors), which may have introduced recall bias. In addition, because no local Turkish normative data or age-specific physiological growth curves are currently available, reference values from In addition, because no local Turkish normative data or age-specific physiological growth curves are currently available, reference values from Tideman et al. [4] and Truckenbrod et al. [15] were used. were used to estimate physiological axial elongation rates. This limitation underscores the need for future studies establishing normative ocular growth data for the Turkish pediatric population.

## 6. Conclusions

This real-world study demonstrated that DIMS spectacle lenses effectively slowed myopia progression and axial elongation within our Turkish pediatric cohort over one year, supporting their real-world effectiveness in pediatric myopia management beyond East Asian populations. Baseline axial length was identified as the strongest predictor of treatment response, while age and gender had a limited influence. These findings highlight the importance of individualized strategies for childhood myopia control and support the use of age-specific physiological growth curves for a more accurate evaluation of treatment outcomes.

## Figures and Tables

**Table 1:** a. Continuous Baseline Characteristics of the Study Population (*n* = 108 eyes). b. Categorical Baseline Characteristics of the Study Population (*n* = 108 eyes).

**a**	
**Variable**	**Mean ± SD (Range)**
Age (years)	9.65 ± 2.18 (6–15)
Baseline SER (D)	−3.01 ± 1.47 (−6.37 to −0.50)
Baseline AL (mm)	24.35 ± 1.06 (22.59–26.56)
**b**	
**Variable**	***n*** **(%)**
Gender (Female/Male)	56 (51.9%)/52 (48.1%)
Age group (<10/≥10 years)	68 (63.0%)/40 (37.0%)
Baseline AL group (Moderate/High)	60 (55.6%)/48 (44.4%)

Note: Values are presented as mean ± standard deviation (SD) or number (percentage) of eyes. All participants were treated with Defocus Incorporated Multiple Segments (DIMS) lenses. Only descriptive statistics are provided, as the study included a single treatment group.

**Table 3:** a. Results of Generalized Estimating Equations (GEE) for SER Success. b. Results of Generalized Estimating Equations (GEE) for AL Response.

**a**										
**Variable**	**B**	**Std. Error**	**95% CI Lower**		**95% CI Upper**		**Wald χ^2^**		**df**	***p*** **-Value**
Gender	−1.441	0.5749	−2.568		−0.314		6.284		1	0.012
Age group	−0.046	0.5671	−1.158		1.065		0.007		1	0.935
Baseline AL group	1.260	0.5538	0.174		2.345		5.174		1	0.023
**b**										
**Variable**	**B**	**Std. Error**	**95% CI Lower**	**95% CI Upper**	**Wald χ^2^**	**df**	***p*** **-Value**	**Exp(B)**	**95% CI Exp(B) Lower**	**95% CI Exp(B) Upper**
Gender	0.447	0.4072	−0.351	1.245	1.206	1	0.272	1.564	0.704	3.474
Age group	−0.552	0.4138	−1.363	0.259	1.781	1	0.182	0.576	0.256	1.295
Baseline AL group	−1.182	0.4057	−1.977	−0.387	8.487	1	0.004	0.307	0.138	0.679

Note: Generalized Estimating Equations (GEE) were used to identify predictors of treatment outcomes. For SER success (Table 3a), a binary GEE model was applied, whereas for AL response (Table 3b), an ordinal GEE model was used. B = regression coefficient; OR = odds ratio; CI = confidence interval; SER = spherical equivalent refraction; AL = axial length.

## Data Availability

The data supporting the findings of this study are available from the corresponding author upon reasonable request.
